# Effects of COVID‐19 pandemic on adherence to obstructive sleep apnea therapy: A case report

**DOI:** 10.1002/ccr3.3363

**Published:** 2020-12-01

**Authors:** Samira Naime, Miriam Weiss, Gustavo Nino

**Affiliations:** ^1^ Division of Pediatric Pulmonary and Sleep Medicine Children’s National Hospital Washington District of Columbia; ^2^ Department of Neurology George Washington University School of Medicine and Health Sciences Washington District of Columbia

**Keywords:** children, COVID‐19, CPAP, OSA

## Abstract

Telemedicine and remote monitoring are valuable tools to address inadequate obstructive sleep apnea compliance during the current pandemic.

## Graphical abstract

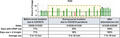

## 1 INTRODUCTION

Obstructive sleep apnea (OSA) is a common condition in adults and children. Treatment of sleep apnea is important particularly in patients with complex medical conditions. Factors affecting adherence to positive airway pressure (PAP) therapy have been described; however, it is unclear how a drastic change in schedules due to social distancing during a pandemic may affect PAP adherence. We present a case of a teenage patient with OSA on PAP therapy whose compliance changed dramatically after the state lockdown started, and has been successfully reversed with telemedicine.

Obstructive sleep apnea (OSA) is common in pediatric patients; it is seen in approximately 1%‐4% of otherwise healthy children.^1^ The first‐line treatment of OSA in children is surgical; nevertheless, the use of positive airway pressure (PAP) is recommended for those with persistent OSA.^1^, ^2^ Adherence to PAP therapy depends on a variety of factors, including patient characteristics, psychosocial factors, educational, and behavioral support.^3^, ^4^ In the pediatric population, there is a lack of data on PAP adherence; however, factors that hinder and improve PAP use have been described.^1^, ^2^ Factors hampering PAP use include adolescent age, personality, and parental education.^1^, ^2^ It is unclear whether a pandemic will affect the adherence to PAP therapy in patients with OSA. We present a case of a 15‐year‐old male patient with OSA on PAP therapy and good adherence to therapy until the start of social isolation, and the cessation of in‐person school due to COVID‐19 pandemic.

## 2 REPORT OF CASE

**FIGURE 1:**
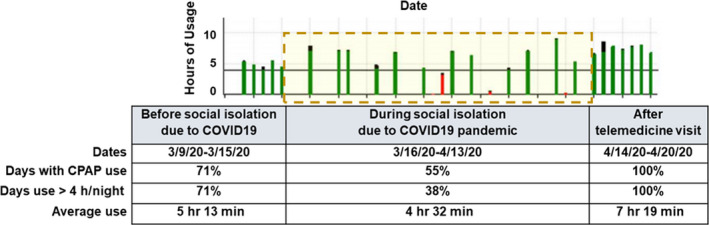
A visual representation of CPAP therapy in our patient. The figure shows the trend of CPAP use before the lockdown, during the lockdown (dashed yellow box) and after telehealth clinic visit. (X‐axis: Date of CPAP use in 2‐wk interval. Y‐axis: hours of CPAP usage. Red bar: CPAP use ≤4 h/night. Green bar: CPAP use ≥4 h/night.) The table details the compliance data downloaded from the patient's CPAP for the period of time illustrated in the graph. CPAP, continuous positive airway pressure

A 15‐year‐old African American male patient with obesity, prediabetes, chronic rhinitis, mild intermittent asthma, moderate obstructive sleep apnea on continuous positive airway pressure (CPAP), status postadenotonsillectomy, and laparoscopic sleeve gastrectomy presented for follow‐up in sleep clinic. The patient was first diagnosed with severe OSA at 9 years of age with an overnight polysomnogram (PSG) showing an obstructive apnea‐hypopnea index (OAHI): 10.3/h. Following the diagnosis, he underwent evaluation by an otolaryngologist and had adenotonsillectomy, after which his symptoms and his repeat PSG showed improvement. The patient was seen in our sleep clinic for initial evaluation 2 years later, due to recurrence of symptoms consistent with sleep apnea along with significant weight gain. His PSG at the time showed severe OSA with OAHI: 30.9/h and CPAP therapy was started. The patient has been on CPAP of 10 cm H_2_O and has had a good adherence to therapy with overall use of 71% of the nights and at least 4 hours of use 71% of the days (Figure 1). Of note, the patient was concomitantly being managed by a multidisciplinary clinic specialized in childhood obesity. He had been assessed by a dietician, endocrinologist, psychologist, and other specialists.

The follow‐up visit in the sleep medicine clinic was conducted via telehealth due to the COVID‐19 pandemic. We met with the patient and parent via secure video conference. The patient was alert, oriented, and appeared to have normal affect without evidence of mood disturbances. He had no complaints concerning his sleep or PAP therapy, except for few instances several weeks before the lockdown when he did not use his CPAP due to nasal congestion. However, after looking at the trend of CPAP use, it was clear that the patient's adherence was dramatically reduced when the COVID‐19 pandemic was announced and social distancing was reinforced. We reviewed with the family the CPAP usage cloud‐based data, obtained remotely, and highlighted how the adherence changed before and during the lockdown. The parent was unaware of the trend and was appreciative that she had this pointed out to her. After that, we discussed once again the morbidities of OSA, the importance of a good sleep routine, and adherence to PAP therapy given the patient's health history. We checked on the patient's compliance 1 week following the telehealth visit and noticed that his adherence to therapy had increased to 100% (Figure 1). Notably, the patient's sleep duration has also increased in comparison with when he was still attending school.

## 3 DISCUSSION

The COVID‐19 pandemic has resulted in a worldwide lockdown as a measure to control the spread of the disease. A statewide lockdown is unprecedented action in the recent era, and its effect on the different medical illnesses is unknown. The need to social distance and self‐isolation has resulted in decreased access to health care and interrupted multiple clinic visits. Patients with chronic illness who require close monitoring and various medications are at a higher risk during a lockdown. ^5^ It is likely that such a lockdown not only affects medication compliance, but may also affect CPAP adherence, as noted in our patient.

The change in life patterns can be stressful to a lot of people, and stress has been associated with worsening of several underlying illnesses.^5^, ^6^ Stress can also affect medication adherence in patients with chronic illness.^7^ Adolescents are at high risk of conflicts with parents and are susceptible to disruptive behaviors, especially in stressful situations which can further exacerbate medication adherence and likely CPAP adherence. In our case, the lack of in‐person school routine due to COVID‐19 could have contributed to poor compliance as well. Given the fact that untreated OSA increases morbidity in patients with moderate‐to‐severe OSA, it is crucial to maintain therapy at times like these.^1^, ^8^

It is worth noting that, during the lockdown, our adolescent patients have sleep schedules that reflect their biologically delayed circadian rhythm. Our patient had a short duration of CPAP use when he was attending school, which could be partly due to the early school start time. This brings to light the importance of delaying school start times to allow for increased sleep duration, improvement in performance, and enhancement of health outcomes in middle school and high school students.^9^ We think that increased sleep duration due to the lack of early school times, and the presence of more parental supervision during the COVID‐19 lockdown, may have positively influenced the usage of CPAP in our patient after the telemedicine visit.

Telemedicine has been used in patients with OSA and has shown improvement in CPAP therapy adherence in some studies.^10^ Telemedicine provides an alternative to clinic visit during the lockdown. It also decreases the exposure of patients with chronic disease to the hospitals and emergency rooms. There are a lot of services that sleep specialists can offer patients with OSA on CPAP therapy via telemedicine. Some of the benefits of telemedicine include the potential “real‐life” home assessment of the sleep environment, CPAP machine, and the mask fit during the visit. Conversely, telemedicine has some limitations including the lack of physical examination, and the technological difficulties encountered by some families with limited Internet and/or computer access. Practicing medicine during COVID‐19 lockdown showed that the benefits of telemedicine are numerous, and it is an alternative we must consider, especially during lockdown or when access to healthcare is limited. The effect of telemedicine on PAP compliance may be more pronounced in those particular situations as highlighted in our case.

In summary, sleep providers need to be aware of the potential effects of COVID‐19 and social isolation, along with the disruption of daily schedules and sleep routines, on CPAP compliance, particularly in adolescents. In addition, nonemergent sleep visits are being postponed in many settings due to limited healthcare resources available during the COVID‐19 pandemic. Our case shows that telemedicine is a potentially effective alternative for early identification and intervention, and should be considered to prevent inadequate OSA treatment during the current pandemic, and future emergent conditions that may require social distancing.

## CONFLICT OF INTEREST

No conflict of interest available.

## AUTHOR CONTRIBUTIONS

Naime S: wrote the manuscript; Weiss M: involved in data gathering and final revision; Nino G: performed major editing of the manuscript and figure design.

## ETHICAL APPROVAL

The authors have reviewed Wiley's ethical guidelines and abide by it.

## INFORMED CONSENT

Consent for publication was obtained from the patient and parent.
